# Application of hybrid SFLA-ACO algorithm and CAM softwares for optimization of drilling tool path problems

**DOI:** 10.1007/s42452-022-05271-x

**Published:** 2023-01-20

**Authors:** Nasir Mehmood, Muhammad Umer, Umer Asgher

**Affiliations:** 1 Business and Engineering Management Department/Sir Syed, CASE Institute of Technology, Islamabad, Pakistan; 2 Quality Assurance & NUST International Office Directorate (QA & NIO Dte), National University of Science and Technology (NUST), Islamabad, Pakistan

**Keywords:** Industrial management, Manufacturing management, Artificial intelligence, Optimization, Metaheuristic algorithms, Computer aided manufacturing

## Abstract

**Abstract:**

In drilling process almost seventy percent time is spent in tool switching and moving the spindle from one hole to the other. This time travel is non productive as it does not take part in actual drilling process. Therefore, this non productive time needs to be optimized. Different metaheuristic algorithms have been applied to minimize this non productive tool travel time. In this study, two metaheuristic approaches, shuffled frog leaping algorithm (SFLA) and ant colony optimization (ACO) have been hybridized. In industry, the CAM softwares are employed for minimization of non productive tool travel time and it is considered that the path obtained by using the CAM softwares is the optimized path. However this is not the case in all problems. In order to show the contribution of the SFLA-ACO algorithm and to prove that results achieved through CAM softwares are not always optimized, hybrid SFLA-ACO algorithm has been applied to two drilling problems as case studies with the main objective of minimization of non productive tool travel time. The drilling problems which are taken from the manufacturing industry include ventilator manifold problem and lift axle mounting bracket problem. The results of hybrid SFLA-ACO algorithm have been compared with the results of commercially available computer aided manufacturing (CAM) software. For comparison purpose, the CAM softwares used are Creo 6.0, Pro E, Siemens NX and Solidworks. The comparison shows that the results of proposed hybrid SFLA-ACO algorithm are better than commercially available CAM softwares in both real world manufacturing problems.

**Article highlights:**

- Different optimization techniques are being used for optimization of drilling tool path problems. In this paper two techniques SFLA and ACO has been combined to form a hybrid SFLA-ACO algorithm and has been applied to the real world industrial problems.
- Two real world problems have been taken from the local manufacturing industries. In both the problems the objective is to optimize the tool traveling time through hybrid SFLA-ACO and compare it with CAM software.
- Four CAM softwares have been used for comparison purpose. The problems undertaken are solved through these CAM software and compared with the results of hybrid SFLA-ACO results. As result of comparison it is found that in both the problems the performance of hybrid SFLA-ACO algorithm remains outclass. This signifies that results of CAM software in case of optimization of drilling tool path are not always optimal and these can be improved by using different optimization techniques.

## Introduction

Drilling is circular hole making process. Drilling operation is used for first time hole creating in the work piece. Reaming, boring, counter boring, counter sinking, spot facing, and tapping all are different drilling processes. The importance of drilling process can be evaluated by the fact that it is used in almost all manufacturing industries and it alone constitutes twenty five percent of all machining processes. Drilling process includes drilling the hole, tool switching and tool travelling between the hole locations. The tool switching and the tool traveling to different holes constitute 70% of the drilling time [1]. This 70% of the drilling time does not contribute to actual drilling process. The tool travelling time is nonproductive as it does not add any value to the work piece. Therefore, this nonproductive time needs to be optimized.

In order to reduce this non productive time, hybrid SFLA-ACO has been applied to six benchmark problems in [2] which has proven the efficacy and better performance of hybrid SFLA-ACO algorithm as compared to its predecessors, SFLA and ACO. The originality and novelty of hybrid SFLA-ACO algorithm demand its validation through application to practical problems of manufacturing industry. Therefore it becomes necessary to apply SFLA-ACO algorithm to real world drilling problems to prove its originality and efficacy. So in this paper, hybrid SFLA-ACO algorithm will be applied first time to lift axle mounting bracket problem and ventilator manifold problem to show its originality and prove its efficacy. The efficacy and performance of hybrid SFLA-ACO will be checked by applying it to these two real world manufacturing problems, taken from local industry, for optimization of tool travel time. The results achieved through hybrid SFLA-ACO algorithm will be compared with four commercially available CAM softwares to evaluate the performance of hybrid SFLA-ACO algorithm.

Drilling problems can be classified as rectangular, circular, multi-surface, path constraint problems, or combination of any of these as per the location of holes. In rectangular problems, the holes are located in the rows and column shape and are very often equidistant. In circular drilling problems, holes are located on circular paths of different diameters. There are some problems in which spindle cannot move directly from one hole to the other hole due to some drawing feature in the way. This type of problems is known as path constraint problems. In some problems there are more than one surface involved which are not on same horizontal plane. This type of problems is called as multi surface drilling problems. Drilling costs are evaluated in terms of time taken in drilling process and the tool wear. As actual drilling, tool switching and tool travelling constitutes the drilling process, therefore time taken by each process is summed up for complete drilling process. Tool traveling costs are considered as a function of the distance between holes. Mainly there are two types of distances are used in the literature, Euclidean distance and the rectilinear distance. Euclidean distance is used when spindle can move in both x and y axis at a time. Rectilinear distance is used when spindle can move only in one direction at a time. Euclidean and rectilinear distances are calculate as following

1 $$ d_{euclidean} = \, \surd (x1 - x2)^{2} + (y1 - y2)^{2} $$

2 $$ d_{rectilinear} = \, |x1 - x2\left| + \right|y1 - y2| $$

After the introduction in first section, a brief literature review is presented in the second section. The third section provides the brief account of the SFLA and ACO. It also explains the hybrid SFLA-ACO algorithm. In Sect. 4 and 5, two real world industrial problems have been undertaken for optimization of drilling tool path. The Sect. 6 presents the conclusion of the case studies.

## Literature review

CAM softwares have the provision for shortest drilling tool path. This shortest drilling tool path provided by the CAM software is considered as the optimal tool path. Whereas this in not true in all the cases. This is proven from the literature as in many cases the results of metaheuristic approaches have been found better when compared with those of CAM softwares.

Medina-Rodriguez et al. [3] has applied ACO metaheuristics on a PCB and the results achieved through ACO are compared with the results of MasterCAM and it is found that drilling time achieved by ACO is less than the time taken by MasterCAM. In [4], Montiel-Ross et al. applied ACO for optimization of circular pattern drilling problems. ACO has improved the results when compared with MasterCAM results showing that ACO is superior. Nabeel et al. [5] used GA for optimization of drilling tool path optimization problems. Authors have compared the results of GA and ArtCAM showing that GA has produced better results. GA has also been used by Pezer et al. [6] on a circular pattern drilling holes problem. The results produced by GA have outperformed the results of WinCAM, CAMConcept and Catia V5. Nguyen et al. [7] have used two metaheuristcs approaches, ACO and GA. The results achieved are them compared waith cncKad Program. Comparison shows that both ACO and GA has better performance. In [8] Saravanan has applied GA on a drilling multi holes problem. The results achieved through GA were better than results of Autodesk Inventor. Garcia et al. [9] has applied Discrete Teaching Learning Based Optimization (DTLBO) to drilling multi holes problem for optimization of tool travel time. The commercially available software CAMotics is also used on the same problem. When the results of DTLBO and CAMotics are compared, it is found that DTLBO has achieved less tool travel time. Khatiwada et al. [10] has applied GA on circular multi holes drilling problem. NCplot is also use for the results of the same problem for comparison. The comparison shows that the total length of the tool travel is less in case of GA. This shows that GA has produced better results than NCplot. Yildiz and Ozturk [11] have employed hybrid enhanced genetic algorithm on turning operation for determination of cutting parameters. The proposed two stage approach with design space refinement has shown its effectiveness for test problems taken from literature as well as for turning single and multi objective problems. This is a generalized approach and can be used for solution of drilling, milling and grinding problems. In [12] Betul and Ali Riza have employed relatively new Moth-Flame Optimization (MFO) algorithm on milling problem for optimization of profit rate. The results show that MFO is more effective for optimization of manufacturing problems as compared to Cuckoo Search, ACO, handbook recommendations, feasible direct method, hybrid PSO, GA, and hybrid immune algorithm. In [13] Ali Riza et al. have hybridized swarm based Harris Hawk Optimization (HHO), recently developed by Mirjalili, with Nelder-Mead (NM) algorithm, a direct search method which is used for local search and developed by Nelder and Mead. The H-HHONM is employed on three benchmark problems and is found effective on comparison of results with famous algorithms like GA, ACO, PSO, and SA etc. Ali Riza et al. [14] have used three optimization algorithms, namely, a population base Harris Hawk Optimization (HHO) recently developed by Mirjalili, a grasshopper swarm behavior inspired Grasshopper Optimization Algorithm (GOA) developed by Saremi et al. in 2017, and an evolutionary Multi Verse Optimization (MVO) algorithm for optimization of processing parameters of grinding operation. The results obtained through HHO, GOA, and MVO are compared with the results of well known like GA, and ACO etc. which has proved the efficiency and superiority of the HHO, GOA, and MVO algorithm.

The comparison of metaheuristic approaches and the CAM software in the previous paragraph shows that CAM softwares do not provide the optimal results in all situations. It can also be seen that in most of the cases the results have been improved when metaheuristics algorithms are used. Hence the employment of metaheuristc algorithms for the optimization of drilling tool path is very useful and encouraging.

## Hybrid SFLA-ACO algorithm

This section presents the basics of the SFLA, basics of ACO, and development of the proposed hybrid SFLA-ACO algorithm.

### Basics of SFLA

The idea of SFLA was first given by Eusuff and Lansey [15] and it is a metaheuristic technique. The SFLA provides the global search through shuffling process and local search within memplex by using random approach. In SFLA, local search is an iterative process and it is repeated for a predetermined time after which shuffling process takes place for global optima. Again the local search is performed and the shuffling process takes place until the desired optimal solution is achieved [16, 17]. Economic load dispatch problems [18] can be solved by SFLA. Multi objective power flow problems [19], and project management problems [20] can also be solved by using SFLA.

### Basics of ACO

The basics of ACO have been provided in detail in [2], however for coherence and better understanding, it is briefly explained in next few lines. Dorigo et al. [21] introduced the term ACO and proposed ACO algorithm in 1991 which finds the shortest path through indirect communication of ants called stigmergy. ACO algorithm constructs the solution step by step and it improves the solution after the each iteration. It provides an optimal solution which is based on probability. It can be used both for discrete and continuous problems. In production scheduling, both job shop and flexible job shop scheduling problems can be solved by using ACO [22]. ACO has been applied successfully on travelling salesman problem [23], vehicle routing problem [24, 25], task allocation of heterogeneous unmanned aerial vehicles [26], and open shop scheduling problem [27]. The probability of an ant *k* located at hole *i* moving to the hole *j* is given as

3 $${p}_{ij}^{k}= \frac{{{[\tau }_{ij}]}^{\alpha }{{[\eta }_{ij}]}^{\beta }}{{\sum }_{l \in {N}_{i}^{k}} {{[\tau }_{il}]}^{\alpha }{{[\eta }_{il}]}^{\beta }}$$

Where, $\mathrm{\tau ij}$ represents the number of pheromone trails, $\mathrm{\eta ij}$ represents heuristic information, and *α* and *β* are parameters that control the pheromone quantity and heuristic information, respectively.

### Development of proposed hybrid SFLA-ACO algorithm

Hybrid algorithms [28] have been used in literature for production planning and control. In hybrid SFLA-ACO algorithm, SFLA and ACO algorithms have been hybridized to improve the performance by improving the accuracy of the results. The working of hybrid SFLA-ACO has been explained in [2] in detail. A brief account is given below to understand the flow of algorithm.

**Fig. 1:**
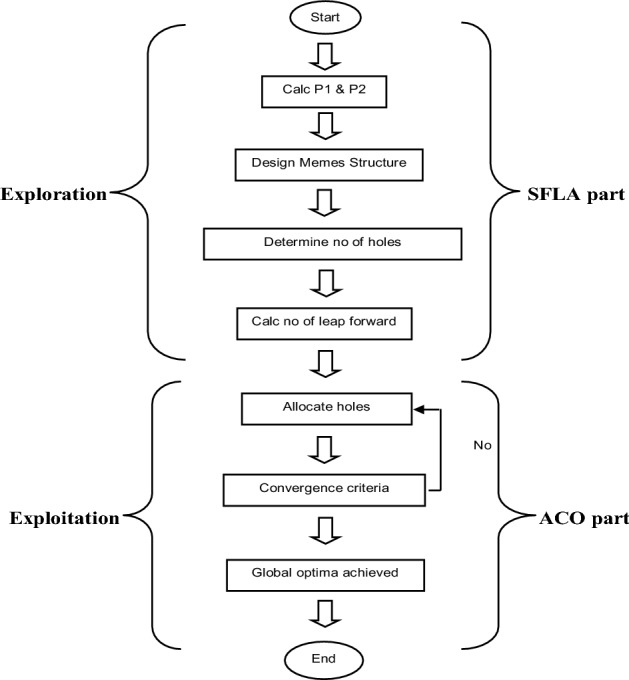
Flow diagram of proposed hybrid SFLA-ACO [2]

The exploration of the design space is carried out by designing the different memeplex structures which is the strong area of SFLA. The global optima is based on this exploration by using different memplexes. The local search is carried out by the ACO metaheuristic. The allocation of each hole in the path is done by using Eq. 3. Equation 3 defines the probability of each hole for the next allocation. This probability is the function of the tool travel distance from present hole to all other non allocated holes and the pheromone quantity. In order to avoid the pheromone accumulation which may cause the algorithm to get stuck in the local optima, pheromone evaporation is carried out at an appropriate rate. The pheromone evaporation rate can be calculated with the help of Eq. 3. The probability for nearer hole is more as compared to the farther hole and vice versa keeping in view the relative distances. A complete path is constructed step by step in this way until all the holes are allocated to the path. Once a path is completed a solution is ready. The cost of the tour is calculated and compared with the convergence criterion of the algorithm. If the convergence criterion is satisfied, the algorithm stops as the optimal result has been achieved. If not then again the local search is carried out. This is a repetitive process and is carried out for a predetermined number of iterations until the convergence criterion meets. The flow diagram below as Fig. 1 explains the complete process of the hybrid SFLA-ACO algorithm.

## Case study I—lift axle mounting bracket problem

This section includes the description of lift axle mounting bracket problem, proposed algorithm results and the comparison of results with those of CAM softwares.

### Problem definition

**Fig. 2:**
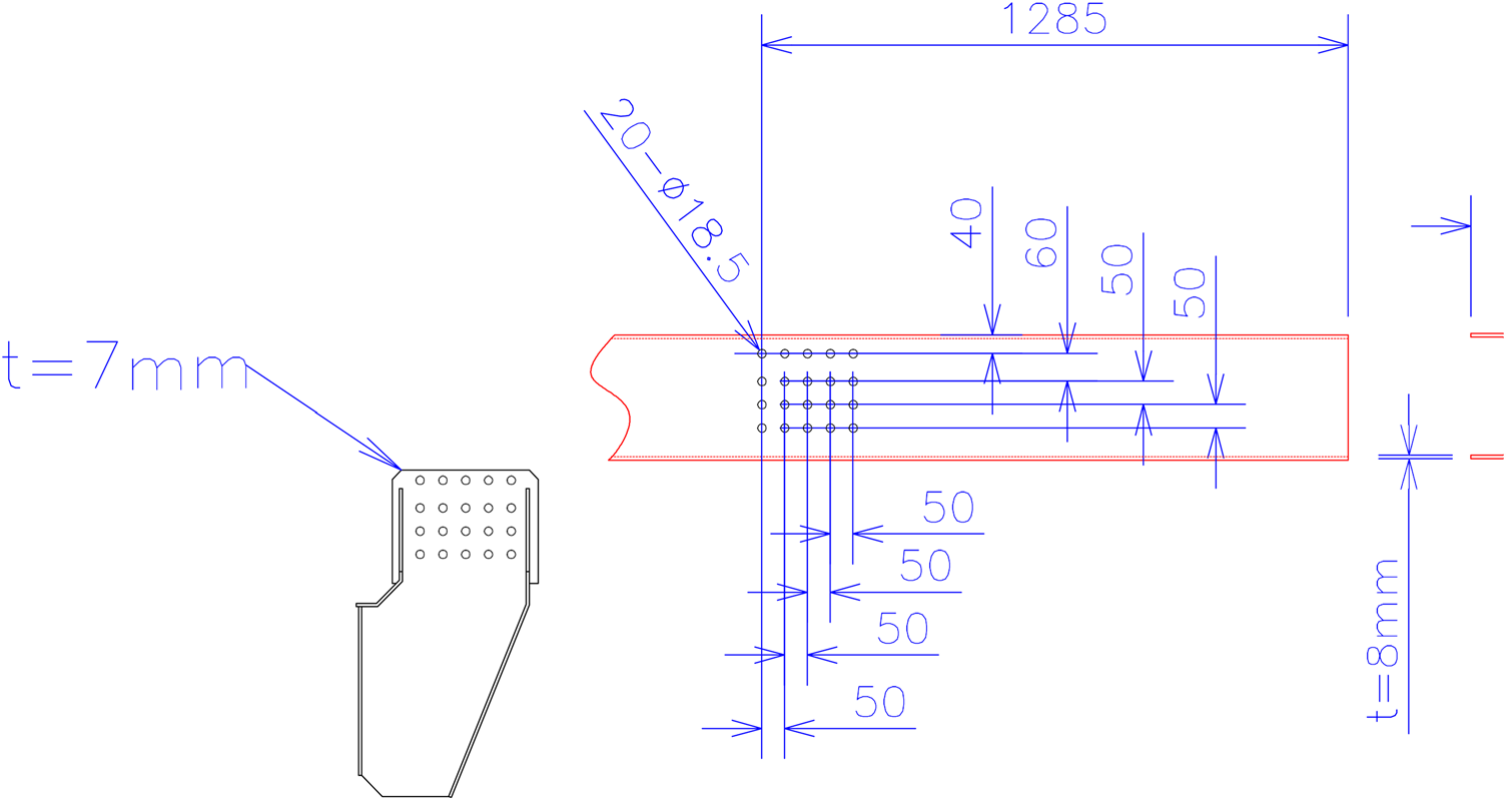
Location and distances of holes in lift axle mounting bracket

There is a concept of lift axle in trucks which have more than two axles. In order to increase the loading capacity of the trucks additional axles are incorporated in the chassis. However, when there is no loading on the truck, the additional axles cause increase in fuel consumption. The wear and tear of the tires also increases. In order to avoid these disadvantages, the additional axle is lifted up and it does not touch the ground. There is a mechanism to lift the axle for which mounting bracket is used. The lift axle and chassis are bolted together through this mounting bracket. The mounting bracket has thickness of 7 mm. There are 20 holes of the same diameter of 18.5 mm on the mounting bracket. Details of holes location and relative distance is shown in Fig. 2 below.

### Problem formulation and results obtained

In lift axle mounting bracket problem involves only one type of holes diameter, therefore tool travel cost is optimized and tool switching costs are considered as zero. The tool travel cost is incurred when the tool moves from its previous hole to the new hole for drilling. Euclidean distance between the holes is used in this study. The total tool travel time is when spindle traverses all the holes and comes to its starting hole. The tool travel time L_de_ when the spindle moves from the present hole location “d” to next hole location “e” can be calculated using the Eq. 1 above.

MATLAB is used for coding of the proposed hybrid SFLA-ACO algorithm. As the metaheuristics are random-based optimization algorithms, therefore the results can vary with each run. The statistical fitness results, e.g. average, maximum, minimum and standard deviation (SD) from ten independent runs have been compared in Table 1 below for axle mount bracket problem. The results show that maximum value reaches to 1181.6 at the most and the minimum value is always 1020 which is the optimal value.

**Table 1:** statistical fitness results for axle mounting bracket problem

Run	Max cost	Min cost	Average cost	No of iterations	Computational time (Sec)
1	1181.8	1020	1117.08	5	3.60
2	1120	1020	1073.9	3	1.50
3	1081.8	1020	1066.3	4	1.54
4	1181.8	1020	1091.3	4	1.54
5	1181.8	1020	1094.5	6	3.80
6	1181.8	1020	1089.4	5	3.60
7	1120	1020	1083.8	7	3.83
8	1081	1020	1066.3	4	1.54
9	1181.8	1020	1104.8	6	3.80
10	1181.8	1020	1099.5	6	3.80

**Fig. 3:**
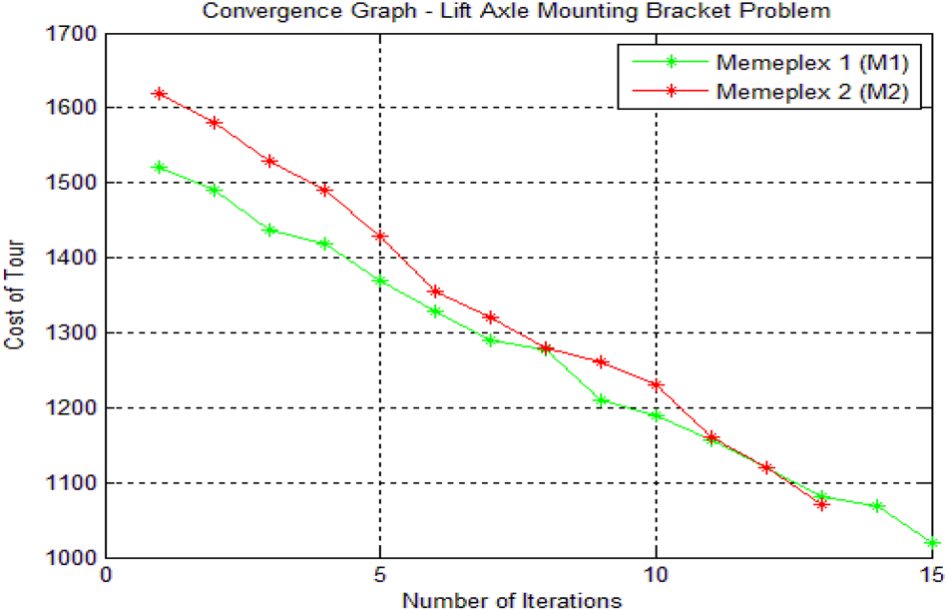
MATLAB graph of the results for lift axle mounting bracket problem

The Fig. 3 below shows the results obtained in form of the convergence graph.

In Fig. 3, the x-axis and the y-axis indicate the number of iterations and the cost of the tours respectively. Two memeplexes (M1 and M2) were used. In M1, the cost of the tour started at 1520 mm and ended at 1020 mm after a total of 15 iterations. In M2, the cost of the tour started at 1620 mm and ended at 1070 mm. The optimized cost of the tour for the lift axle mounting bracket problem is 1020 mm and the optimal sequence by using proposed hybrid algorithm is [1, 8, 9, 16, 17, 18, 15, 10, 7, 6, 11, 14, 19, 20, 13, 12, 5, 4, 3, 2, and 1], which has been obtained through memeplex 1 (M1).

### Comparison of results and discussion

**Fig. 4:**
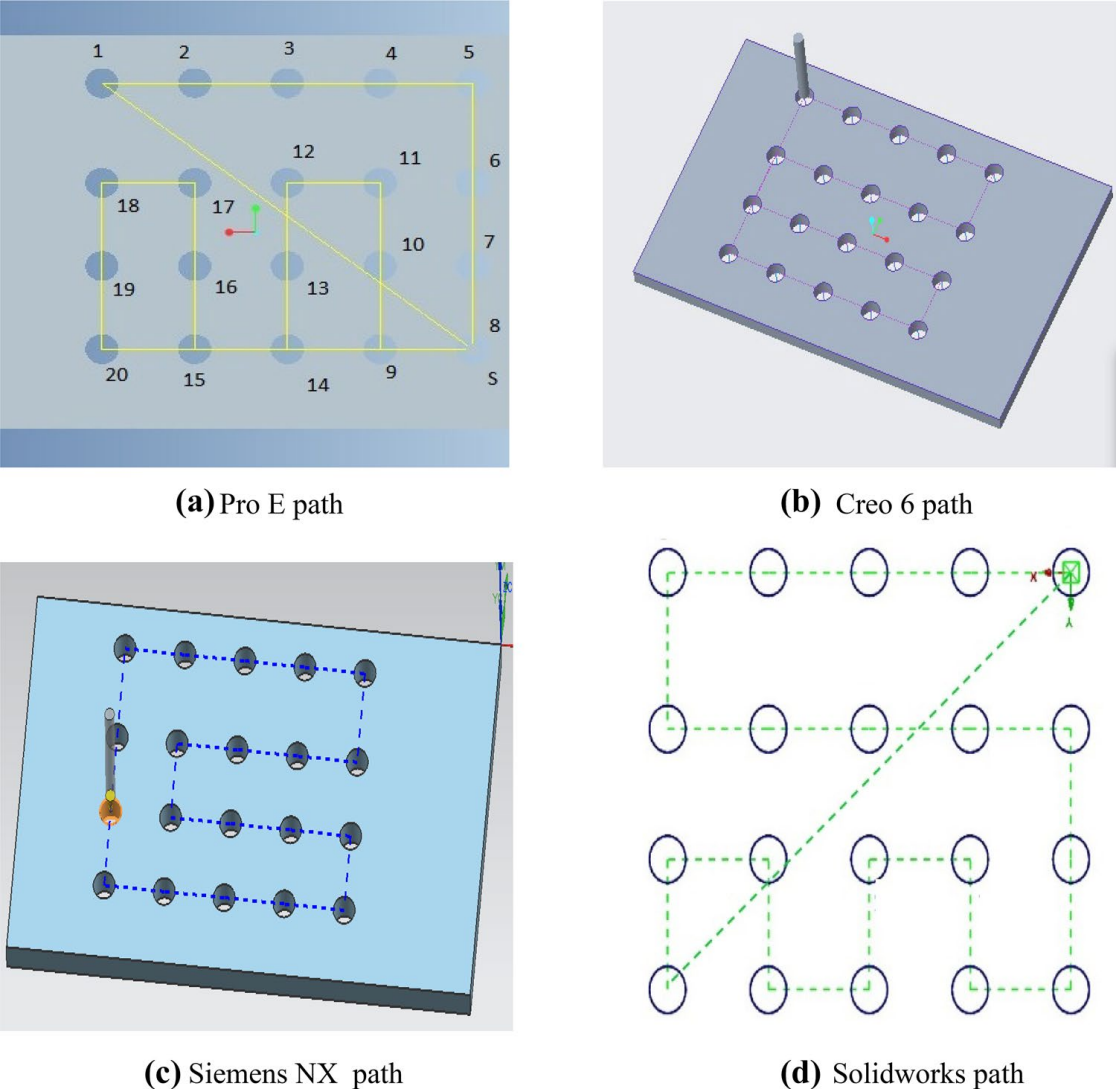
Optimal paths through CAM softwares. **a** Pro E path **b** Creo 6 path **c** Siemens NX path **d** Solidworks path

A comparison has been done between the results of the proposed hybrid algorithm and the results of CAM softwares Pro E, Creo 6.0, Siemens NX, and Solidworks. The paths obtained through Pro E, Creo 6.0, Siemens NX, and Solidworks are shown in Fig. 4 below.

A comparison of the performance with regard to optimal path sequence and its related traveling cost obtained through Pro E, Creo 6.0, Siemens NX, Solidworks, and the proposed hybrid algorithm is given in Table 2 below.

**Table 2:** Comparison of results

Method	Optimal path	Optimal tool travel cost	Relative performance (%)
Pro E	17, 4, 5, 12, 13, 20, 19, 18, 17, 16, 15, 14, 11, 10, 9, 8, 7, 6, 5, 2, 1, 8, 9, 16, 17	1416.12	61.16
Creo 6.0	1, 2, 3, 4, 5, 10, 9, 8, 7, 6, 11, 12, 13, 14, 15, 20, 19, 18, 17, 16, 1	1120	90.20
Siemens NX	2, 1, 8, 9, 16, 17, 18, 15, 10, 7, 6, 11, 14, 19, 20, 13, 12, 5, 4, 3, 2	1020	100
Solidworks	20, 13, 12, 5, 4, 6, 11, 14, 19, 18, 17, 16, 15, 10, 9, 8, 7, 2, 1, 20	1216.12	80.78
Hybrid SFLA-ACO algorithm	1, 2, 3, 4, 5, 10, 9, 8, 7, 12, 13, 14, 15, 20, 19, 18, 17, 16, 11, 6, 1	1020	100

As shown in the Table 2 above, the total nonproductive traveling by the spindle calculated by using Pro E, Creo 6.0, Siemens NX, Solidworks and the proposed hybrid SFLA-ACO algorithm is 1416.12 mm, 1120 mm, 1020 mm, 1216.12 mm, and 1020 mm respectively. From these results, it is clear that proposed hybrid SFLA-ACO algorithm and Siemens NX have produced the best results. If we calculate the relevant performance, then we compare all the results with the best result (1020 mm). The results of the Pro E is 38.84% higher than the best results which means its performance is 38.84% percent less than the best and it comes out to be 61.16%. The result obtained by Creo 6 is 9.8% in the higher side as compared to the best which means that the performance of the Creo 6 is 90.20%. The result obtained using Solidworks is 1216.12 mm which is 19.22% higher than the best. It means its performance is 80.78%. The proposed hybrid SFLA-ACO algorithm has improved the tool travel time as compared to the results obtained through Pro E by 38.84%, Creo 6 by 9.8%, and Solidworks by 19.22%. Hence, the proposed hybrid algorithm has outperformed the CAM softwares.

## Case study II—ventilator manifold problem

This section presents description of the problem, application of the proposed hybrid algorithm and CAM software, and comparison of their results.

### Problem description

**Fig. 5:**
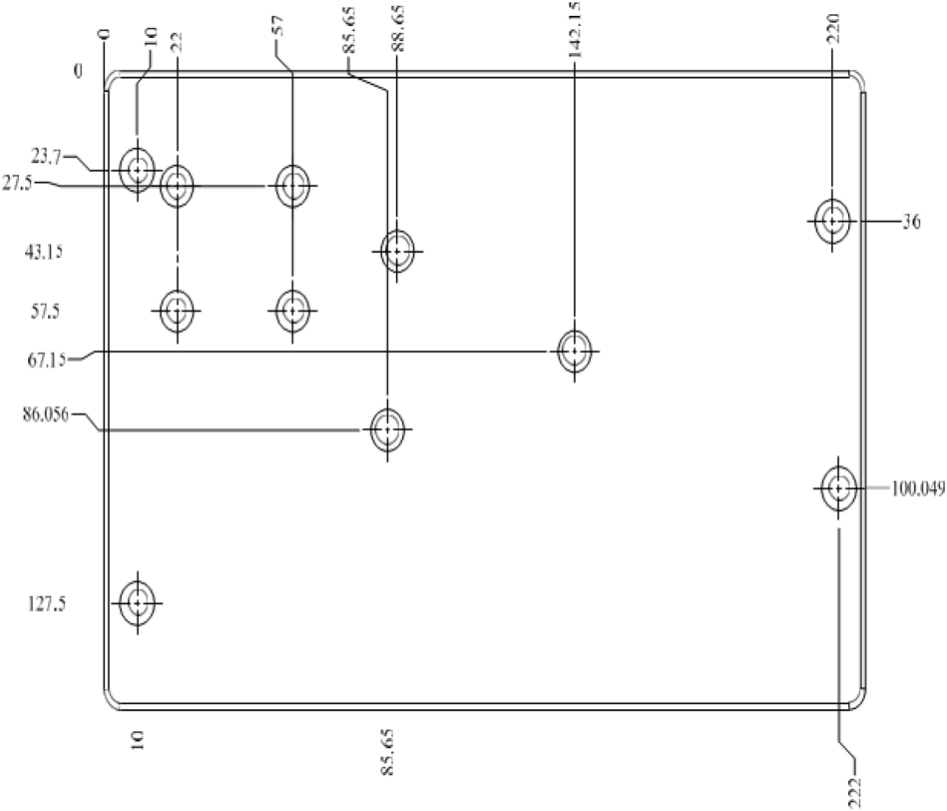
Holes location and distances of top plate of manifold

Drilling of multi-holes in ventilator main manifold problem has been taken as a case study from the bio-medical equipment manufacturing industry. This manifold is a part the ventilators locally manufactured and used for COVID 19. The thickness of manifold plate is 8 mm. There are total 11 numbers of holes on the manifold plate. All holes are of the same diameter (Ø12), therefore there is no requirement for tool switching. The location of each hole and its relative distance has been shown in the Fig. 5 below.

### Problem formulation and results obtained

The drawing shows that there are same diameter holes on the manifold. Therefore there is no need of tool switching. Only the tool travel time is considered for optimization. The tool travel cost is proportional to the tool traveling time between the holes. Euclidean distance between the holes is used in this study. The total tool travel time is when spindle traverses all the holes and comes to its starting hole. The tool travel time L_de_ when the spindle moves from the present hole location “d” to next hole location “e” can be calculated using Eq. 1.

The proposed hybrid algorithm is coded in MATLAB. As the metaheuristics are random-based optimization algorithms, therefore the results can vary with each run. The statistical fitness results, e.g. average, maximum, minimum and SD from ten independent runs have been compared in Table 3 below for axle ventilator manifold problem. The results show that maximum value reaches to 1189.9 at the most and the minimum value is always 654.72 which is the optimal value.

**Table 3:** statistical fitness results for ventilator manifold problem

Run	Max cost	Min cost	Average cost	No of iterations	Computational time
1	1070.5	691.52	820.5	80	13.27
2	1089.9	695.43	834.6	74	12.87
3	1090.7	654.72	810.8	89	18.39
4	1080.6	696.54	796.7	82	15.24
5	1120.4	695.43	815.3	84	16.09
6	1106.4	682.50	790.9	92	19.23
7	1170.5	695.43	812.7	70	12.20
8	1070.5	654.72	795.9	90	18.89
9	1189.9	702.47	841.7	85	16.20
10	1189.9	667.31	809.9	78	13.15

**Fig. 6:**
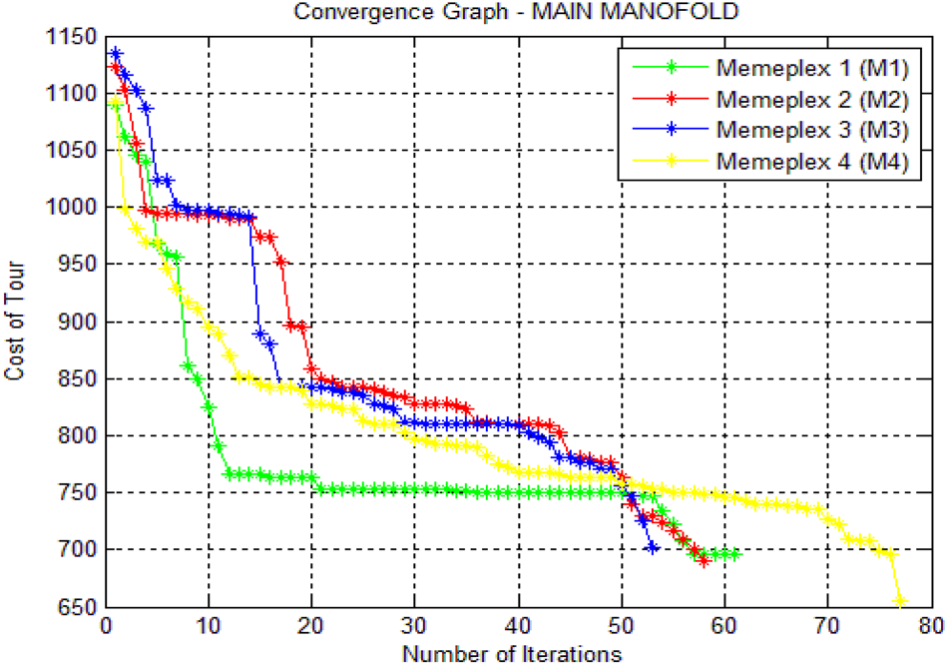
MATLAB graph of the results for manifold problem

The results of optimal tool travel distance obtained are shown in the form of a convergence graph as Fig. 6.

In Fig. 6, the x-axis and the y-axis indicate the number of iterations and the cost of the tours respectively. Four memeplexes (M1, M2, M3 and M4) were used. In M1, the cost of the tour started at 1080.6 mm and ended at 690.4 mm after a total of 61 iterations. In M2, M3, M4 the cost of the tour started at 1125.4 mm, 1140.2, 1095.6, and ended at 679.8 mm,700, 654.7 respectively. The optimized cost of the tour for the ventilator manifold plate problem is 654.72 mm and the optimal sequence by using proposed hybrid algorithm is 1, 3, 4, 2, 7, 9, 11, 10, 8, 6, 5, 1 which has been obtained through memeplex 4 (M4).

### Comparison of results and discussion

**Fig. 7:**
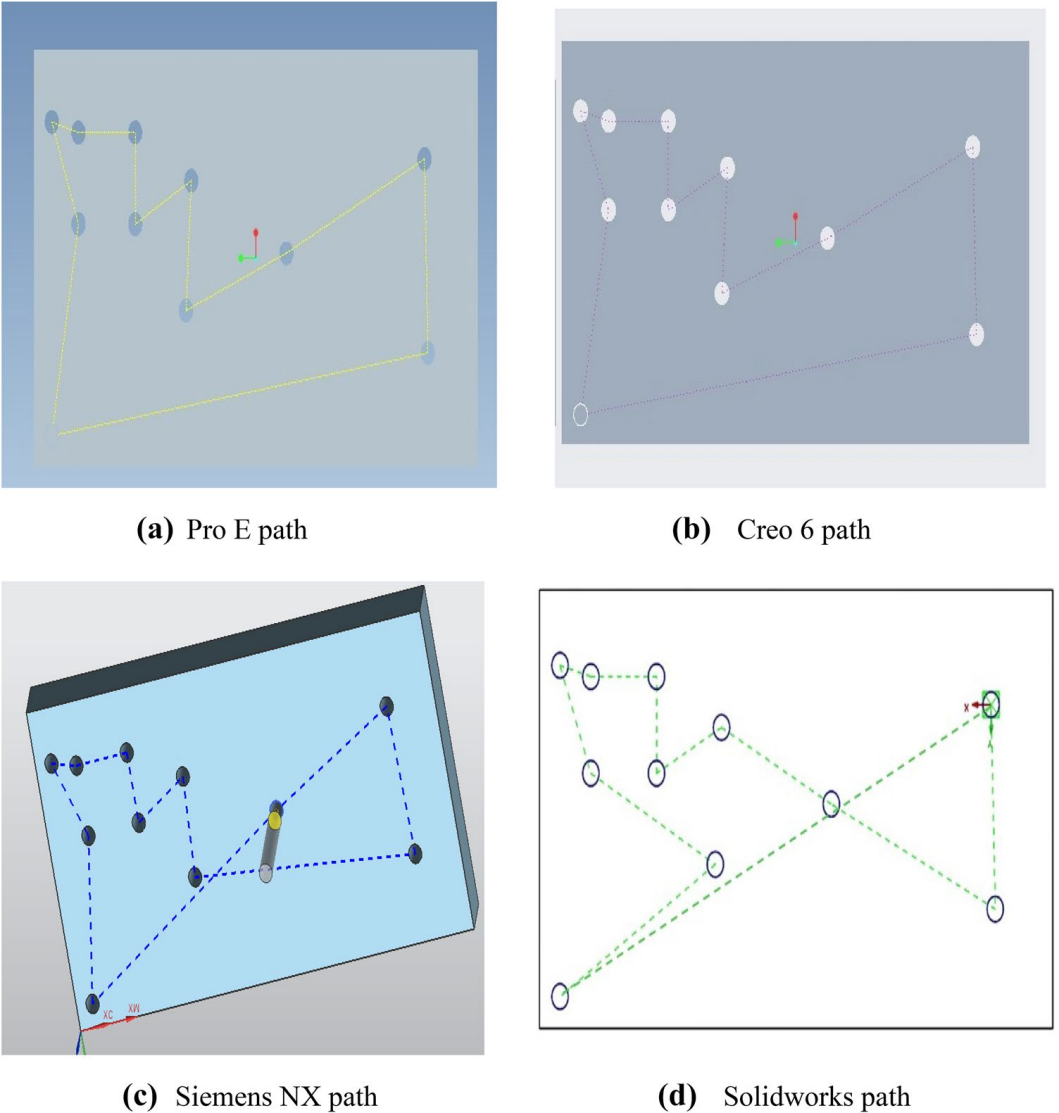
Optimal Paths through CAM softwares. **a** Pro E path **b** Creo 6 path **c** Siemens NX path **d** Solidworks path

For comparison, CAM softwares Pro E, Creo 6.0, Siemens NX, and Solidwork are used to obtain the shortest path. The paths obtained through Pro E, Creo 6.0, Siemens NX, and Solidworks are shown in Fig. 7 below.

Table 4 shows a comparison of the performance with regard to optimal path sequence and its related traveling cost obtained through Pro E, Creo 6.0, Siemens NX, Solidworks, and the proposed hybrid algorithm.

**Table 4:** Comparison of results

Method	Optimal path	Optimal tool travel cost	Relative performance (%)
Pro E	1, 3, 5, 6, 7, 8, 9, 10, 11, 2, 4, 1	675.88	96.77
Creo 6.0	1, 3, 5, 6, 7, 8, 9, 10, 11, 2, 4, 1	675.88	96.77
Siemens NX	9, 10, 11, 7, 8, 6, 5, 3, 1, 4, 2, 9	687.24	95.04
Solidworks	1, 3, 5, 6, 8, 9, 11, 10, 2, 7, 4, 1	746.76	85.95
Hybrid SFLA-ACO algorithm	1, 3, 5, 6, 8, 10, 11, 9, 7, 2, 4, 1	654.72	100

As shown in the Table 4 above, the total nonproductive traveling by the spindle calculated by using Pro E, Creo 6.0, Siemens NX, Solidworks and the proposed hybrid SFLA-ACO algorithm is 675.88 mm, 675.88 mm, 687.24 mm, 746.76 mm, and 654.72 mm respectively. From these results, it is clear that proposed hybrid SFLA-ACO algorithm has produced the best results. If we calculate the relevant performance, then we compare all the results with the best result (654.72 mm). The results of the Pro E and Creo 6 are same and 3.23% higher than the best results which means their performance is 3.23% percent less than the best and it comes out to be 96.77%. The result obtained by Siemens NX is 687.24 mm. This result is 4.96% on the higher side as compared to the best which means that the performance of the Siemens NX is 95.04%. The result obtained using Solidworks is 746.76 mm which is 14.05% higher than the best. It means its performance is 85.95%. The proposed hybrid SFLA-ACO algorithm has improved the tool travel time obtained through Pro E and Creo 6 by 3.23%, Siemens NX by 4.96%, and Solidworks by 14.05%. Hence, the proposed hybrid algorithm has outperformed the CAM software.

## Conclusion

This study was carried out for the application of hybrid SFLA-ACO algorithm on real world drilling tool path problems and its comparison with the commercially available CAM software. The results show that the hybrid SFLA-ACO algorithm has been successful. The comparison of results with CAM software show that performance of the hybrid SFLA-ACO algorithm is better than CAM software as in both case studies the results have been improved. The results encourage the use of optimization techniques in industrial problems for better results. Therefore it is recommended hybrid SFLA-ACO algorithm should be applied to the drilling work pieces which are required to be manufactured in bulk quantities so that maximum advantages can be achieved. As a future research, the hybrid SFLA-ACO can be applied to other optimization problems of manufacturing industry like scheduling and assembly line balancing problems etc. The case study problems were single tool optimization problems which are relatively simpler as compared to the multi tool drilling problems which involve the tool switching and precedent constraints. Therefore it is recommended that hybrid SFLA-ACO should be applied to relatively complex drilling problems. In future, another case study can be undertaken in which hybrid SFLA-ACO, famous metaheuristic like GA, PSO etc. and currently used CAM software in industry are applied to the same benchmark problems. In this way, the efficacy and performance of hybrid SFLA-ACO can be checked against contemporary metaheuristics like GA, PSO etc. and currently used CAM software in industry.

## Data Availability

All necessary data is provided in the manuscript. Any additional data required will be made available as and when required.
